# Statin Use and the Risk of Parkinson's Disease: An Updated Meta-Analysis

**DOI:** 10.1371/journal.pone.0152564

**Published:** 2016-03-28

**Authors:** Shuang Bai, Yi Song, Xin Huang, Lidan Peng, Jie Jia, Yu Liu, Hong Lu

**Affiliations:** 1 Department of Neurology, The First Affiliated Hospital of Zhengzhou University, Zhengzhou, Henan, China; 2 Department of Ultrasound, The First Affiliated Hospital of Zhengzhou University, Zhengzhou, Henan, China; 3 Department of Orthopedics, The First Affiliated Hospital of Zhengzhou University, Zhengzhou, Henan, China; University of Manitoba, CANADA

## Abstract

**Introduction:**

In response to the ongoing debate over the relationship between the use of statins and the risk of Parkinson's disease (PD), we performed a systematic review and meta-analysis of observational studies to examine their association.

**Methods:**

We conducted a review of the literature using electronic databases supplemented by a manual search to identify potentially relevant case-control or cohort studies. Summary relative risk (RRs) and 95% confidence intervals (CIs) were calculated using a random-effects model. Sensitivity and subgroup analyses were also conducted.

**Results:**

Eleven studies (five case-control and six cohort) with a total of 3,513,209 participants and 21,011 PD cases were included. Statin use was associated with a lower risk of PD, with a summary RR of 0.81 (95% CI 0.71–0.92). Sensitivity analysis demonstrated the robustness of results. Subgroup analyses showed that neither study design nor study region significantly influenced the effect estimates. However, subgroup studies adjusted for age or sex had a greater inverse association than did unadjusted analyses (age-adjusted RR 0.75, 95% CI 0.60–0.95; age-unadjusted RR 0.86, 95% CI 0.75–0.99 and sex-adjusted RR 0.76, 95% CI 0.59–0.98; sex-unadjusted RR 0.85, 95% CI 0.79–0.92).

**Conclusions:**

Results of this systematic review suggest that statin use is associated with a reduced PD risk. However, randomized controlled trials and more observational studies should be performed before strong conclusions are drawn.

## Introduction

Parkinson's disease (PD) is the second most common neurodegenerative disease and is characterized by several symptoms, including static tremor, bradykinesia, and rigidity as well as abnormal posture and pace. In most cases, these symptoms are related to a marked depletion of dopaminergic neurons in the substantia nigra pars compacta of the basal ganglia and a subsequent malfunction of the nigro-striatal circuitry [1]. The cause of PD remains unknown, but it seems to result from a complicated interplay of genetic and environmental factors affecting numerous fundamental cellular processes, with possible mechanisms from oxidative stress, immunoinflammatory responses, mitochondrial dysfunction, and proteasome dysfunction [2].

Statins are widely prescribed as lipid-lowing agents to prevent and treat coronary heart disease and cerebral vascular disease. They interfere with the formation of isoprenoid intermediates in the biosynthesis of cholesterol by inhibiting the enzyme HMG-CoA reductase, thus providing hypolipidemic effects [3]. However, evidence suggests that statins have additional effects, such as antioxidant and anti-inflammatory effects, leading to the hypothesis that statins may benefit PD patients [4].

Over the past decade, a number of studies have examined the association between statin use and the risk of PD [5–16]. However, these studies have revealed conflicting results with no clear conclusions. Although a meta-analysis on statin use and the risk of PD has been previously published [17], its results are not sufficiently convincing due to a non-comprehensive literature retrieval and a small number of included studies. Another important limitation is that publication bias was observed using the p values of the Begg's (p = 0.03) and Egger's (p = 0.01) tests and based on the fact that the funnel plot did not show a symmetrical funnel shape.

To date, the association between statin use and PD has thus remained unclear. Therefore, we conducted a meta-analysis in order to reach a convincing conclusion on this topic by combining observational studies (case-control or cohort studies) published through 30th June 2015, since randomized controlled trials (RCTs) on this topic have not been performed for ethical reasons.

## Methods

### Search strategy

A computerized literature search was conducted using MEDLINE, PubMed, Embase, The Cochrane Library, Google scholar and Scopus to identify relevant articles through 30th June 2015. We used the following search terms: "HMG-CoA reductase inhibitor(s)", "statin(s)", "lipid-lowering drug(s)", "lipid-lowering agent(s)", "simvastatin(s)", "pravastatin(s)", "atorvastatin(s)", "rosuvastatin(s)", "fluvastatin(s)", "lovastatin(s)", and "pitavastatin(s)". These terms were connected with the Boolean operator "or" and then combined with "Parkinson's disease", "PD", or "Parkinson disease". Meeting abstracts were searched in the ISI Proceedings database, and we also conducted a search using the American clinical trial register and International Clinical Trials Registry Platform for unpublished literature. The reference lists of the retrieved articles and recent reviews on this topic were also reviewed manually to locate additional eligible studies.

### Inclusion and exclusion criteria

The following criteria were used to select the retrieved studies: First, studies had to be case-control or cohort studies examining the use of statins and the incidence of PD in human subjects. Articles were excluded if they were reviews, comments, replies, case reports, experimental studies or meeting abstracts of identified full articles. Second, they had to provide explicit descriptions of statin use, detailed methods for diagnosing PD, and the confounding variables adjusted in the analysis. Finally, if multiple studies reported data from the same population, only the one with largest sample size or most information was included, and the others were excluded.

Two authors independently selected studies by a two-stage process according to the above criteria: screening the titles and abstracts and then assessing the full-text of potential articles for eligibility. Disagreements were resolved through discussion with another researcher.

### Data extraction

Data were extracted independently by three authors using data extraction forms. When data were insufficient or missing, we contacted the study’s authors for additional information. We obtained the following information from each included study: the last name of the first author, publication year, study period, country in which the study was conducted, study design (case-control or cohort study), number of participants and PD cases, description of statin use, methods for diagnosing PD, variables adjusted, effect estimates (adjusted odds ratio [OR], relative risk [RR], or hazard ratio [HR]), and 95% confidence intervals (CIs). Maximally adjusted effect estimates were extracted both overall and in subgroup analyses.

### Assessment of study quality

Three authors assessed the quality of each included study independently using the Newcastle-Ottawa Scale (NOS) [18]. The NOS comprises three domains: selection, comparability, and outcome for cohort studies or exposure for case-control studies. A maximum of four stars are awarded for selection, two stars for comparability, and three stars for exposure/outcome. Scores range from 0 to 9 stars, and studies with a score of greater than 6 stars were considered to be of high quality, 6 stars to be of medium quality, and less than 6 stars to be of low quality. We resolved disagreements by discussion to reach a consensus.

### Statistical methods and assessment of homogeneity

On account of the low incidence of PD, the distinctions among RR, HR, and OR could be ignored [19]. Therefore, we combined case-control and cohort studies and calculated the summary RRs and 95% CIs. We assessed heterogeneity among studies using the Cochrane's Q test and I^2^ statistics. For the Q test, a p value < 0.10 was considered to indicate significant heterogeneity, while for I^2^, a value of 0%–25% represented insignificant heterogeneity, a value > 25% but ≤ 50% represented low heterogeneity, a value > 50% but ≤ 75% represented moderate heterogeneity, and a value > 75% represented high heterogeneity [20]. In cases of moderate heterogeneity, summary RRs were calculated using the random-effects model (DerSimonian and Laird method), which allowed each of the studies in the meta-analysis to estimate a different effect size. We used the results of the original studies from multivariate models with the most complete adjustment for potential confounders.

Publication bias was assessed by both the Begg's rank correlation test and the Egger's linear regression test, with p < 0.10 indicating statistical significance. Additionally, the funnel plot was applied.

Sensitivity analyses and subgroup analyses were performed. We conducted subgroup meta-analyses by the type of study design, the study region, the variables adjusted and the quality of studies.

To examine the association between individual statin use (or long-term statin use) and the risk of PD, we calculated the pooled RRs from studies providing these particular data.

All analyses were carried out using STATA software (version 14.0).

## Results

### Search results

**Fig 1:**
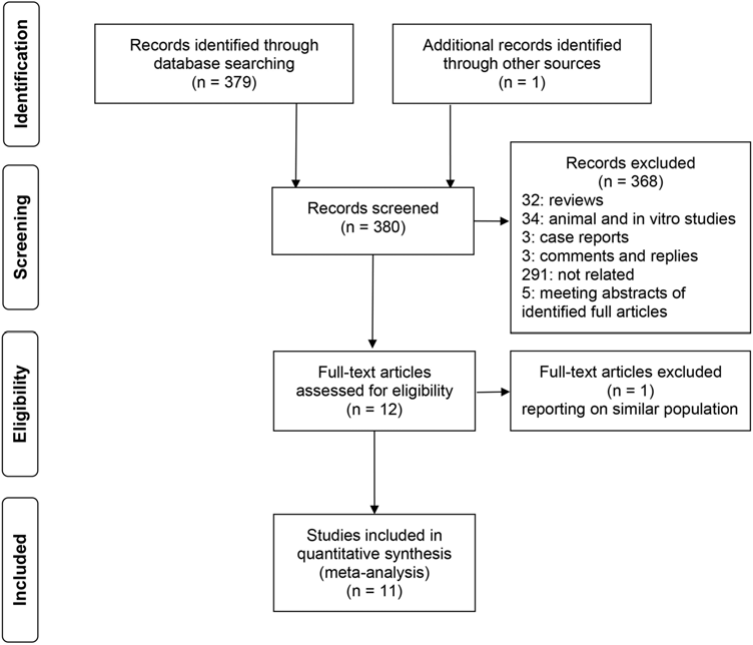
Flow diagram representing the selection process.

We identified 328 articles and 51 meeting abstracts through the computerized search and one additional article through the manual search. However, after reviewing the titles and abstracts of all 380 articles, 368 were excluded because they were either animal experiments, *in vitro* experiments, review articles, case reports, comments, replies, irrelevant studies or meeting abstracts of identified full articles. The full texts of the remaining 12 studies were obtained and assessed according to the eligibility criteria. Finally, 11 articles were included in our meta-analysis, excluding one article that reported data from the same population reported in one of the other 11 [16]. Details of the study selection process and reasons for exclusion are shown in Fig 1.

### Study characteristics

We identified five case-control studies and six cohort studies, which included a total of 3,513,209 participants and 21,011 incident cases of PD (Table 1).

**Table 1:** Characteristics of studies included in the meta-analysis.

First author (publication year)	Country	Study period (year)	Study design	Population	PD cases	Description of statin use ^a^	Variables adjusted ^b^	Quality score (NOS)
Huang (2007) [5]	USA	2002–2004	Case-control	236	124	A	1,2,7,27	7
de Lau (2007) [6]	Netherland	1990–2004	Cohort	6465	87	A	1,2,7	8
Wolozin (2007) [7]	USA	2003–2005	Cohort	1226198	5107	B	9,12,20	6
Sammi (2008) [8]	Canada	1997–2003	Case-control	23780	4756	B	2,18,28	6
Wahner (2008) [9]	USA	2001–2007	Case-control	654	312	A	1,2,3,4,5,7	7
Becker (2008) [10]	UK	1994–2005	Case-control	7274	3637	B	7,8,18,19,21	9
Ritz (2010) [11]	Denmark	2001–2006	Case-control	11582	1931	A	1,2,9,10	7
Hippisley-Cox (2010) [12]	UK	2002–2008	Cohort	2004692	3533	B	1,7,8,17,30	6
Gao (2012) [13]	USA	1994–2006	Cohort	129066	644	A	1,7,8,11,13,14,16,22–26	7
Friedman (2013) [14]	Isreal	2000–2007	Cohort	87971	824	A	2,6,7,11,14,15,16	8
Huang (2015) [15]	USA	1987–2008	Cohort	15291	56	A	1,2,3,7,11,16,23,29	6

PD, Parkinson's disease; NOS, Newcastle-Ottawa Scale.

^a^ A, ever use of statins versus never use of statins; B, current use of statins versus none use of statins.

^b^ 1. age; 2. sex; 3. race; 4. country; 5. education; 6. low socioeconomic status; 7. smoking; 8. body mass index; 9. Charlson index; 10. chronic obstructive pulmonary disease; 11. cardiovascular diseases; 12. dementia; 13. duration of hypercholesterolemia; 14. hypertension; 15. CVA; 16. diabetes; 17. depression; 18. comorbidities; 19. use of fibrates; 20. use of neuroleptic medication; 21. use of hypertension drugs; 22. use of ibuprofen; 23. use of caffeine; 24. use of lactose; 25. use of alcohol; 26. physical activity; 27. low-density lipoprotein cholesterol; 28. use of antipsychotics; 29. average total cholesterol; 30. use of tricyclic antidepressants.

The five case-control studies involved 43,526 participants, including 1,008 statin users among 10,760 PD cases and 3,610 statin users among 32,766 controls. Of the five studies, two reported a reduced risk of PD among statin users [5, 9]. Statin use was confirmed by reviewing medical records in four studies [5, 8, 10, 11] and by self-report in one study [9]. PD diagnosis was ascertained by neurologists in two studies [5, 9] and through medical records in three studies [8, 10, 11]. Of these studies, three studies were conducted in North America [5, 8, 9] and two in Europe [10, 11].

The six cohort studies involved a total of 3,469,683 participants and identified 10,251 PD patients after follow-up periods ranging from 2 to 21 years. Of the 3,469,683 participants, 1,096,147 were statin users. Three studies reported an inverse association between statin use and risk of PD [12, 13, 14]. Statin use was ascertained by reviewing medical records in four studies [6, 7, 12, 14] and by self-report in two studies [13, 15]. PD diagnosis was confirmed by screening for PD signs and a subsequent structural diagnostic workup in one study [6], reviewing medical records in three studies [7, 12, 14], and by multiple approaches (self-report, medical record, death certificate, hospitalization record and physician's confirmation) in two studies [13, 15]. Of these studies, two were conducted in Europe [6, 12], three in the USA [7, 13, 15], and one in Asia [14].

In addition, four studies reported the relationship between individual statin use and the risk of PD, covering simvastatin, atorvastatin, lovastatin, pravastatin, and rosuvastatin [7, 9, 10, 12]. Four studies presented examinations of long-term statin use in relation to risk of PD [9, 11, 13, 14]. The definitions of long-term statin use were at least 4 years, 5 years, or 6 years, respectively.

### Quality of included studies

Ratings of the quality of studies according to the NOS are presented in Table 1. Quality scores ranged from 6 to 9 stars. All studies ensured comparability by adjusting for potential confounders, including age and sex in most studies. For the included cohort studies, the follow-up duration for one study was not long enough (2 years) to detect a sufficient number of PD cases [7]. For the case-control studies, there was good representation of patients, and most controls were selected from the community, with the exception in one study [5].

### Overall analysis

**Fig 2:**
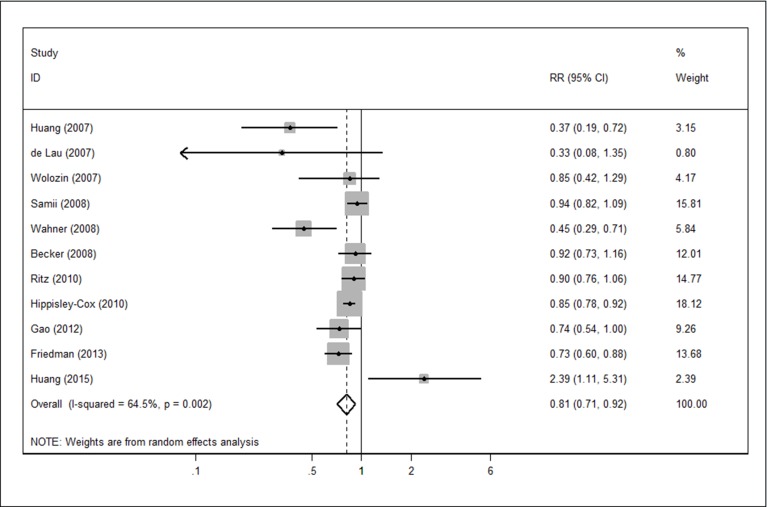
Forest plot in overall analysis.

We combined the 11 studies with a random-effects model, obtaining a summary RR of 0.81 (95% CI 0.71–0.92); that is, there was a 19% reduction in the risk of developing PD for statin users as compared with non-users. Moderate homogeneity was detected among studies (Cochrane Q value = 28.18, p *=* 0.002; I^2^ = 64.5%) (Fig 2).

### Assessment of publication bias

**Fig 3:**
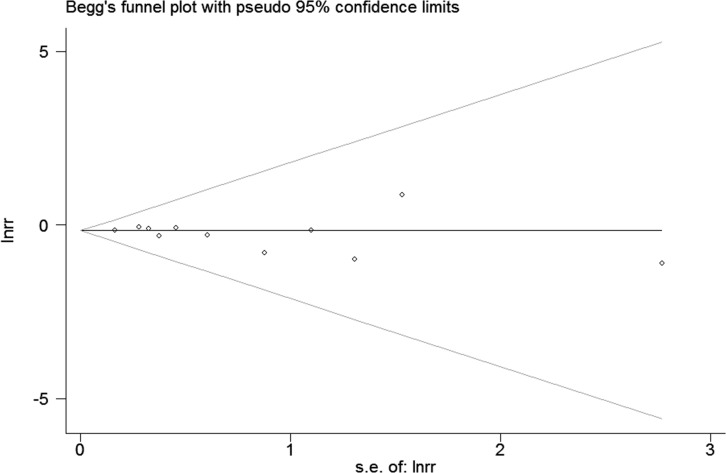
Begg's Funnel plot.

The Egger's test (p = 0.348) and the Begg's test (p = 0.350) provided no evidence for publication bias among studies, and the funnel plot displayed an approximately symmetrical funnel shape (Fig 3).

### Sensitivity and subgroup analyses

Sensitivity analyses were conducted to test the stability of outcomes. We calculated the pooled RRs by excluding one study at a time, and repeated this process 11 times. No significant variation was detected in pooled RRs when excluding any article. The pooled RRs ranged from 0.79 to 0.84, confirming the robustness of the results.

Statin use was found to be associated with a decreased risk of PD in both case-control studies (RR 0.77, 95% CI 0.62–0.97) and cohort studies (RR 0.82, 95% CI 0.68–0.99). When grouped by region, an inverse association was found in the European group (RR 0.86, 95% CI 0.80–0.93) and the Asian group (only one study, RR 0.73, 95% CI 0.60–0.88), while the association was non-significant in the North American group (RR 0.76, 95% CI 0.54–1.08). The pooled RRs of the studies that were adjusted for age or sex presented more significant inverse associations (RR 0.75, 95% CI 0.60–0.95 and RR 0.76, 95% CI 0.59–0.98, respectively) than did studies that were not adjusted (RR 0.86, 95% CI 0.75–0.99 and RR 0.85, 95% CI 0.79–0.92, respectively). The subgroup of high-quality studies showed an inverse association (RR 0.71, 95% CI 0.59–0.87) between statin and risk of PD, but this association was non-significant in the medium-quality group (RR 0.83, 95% CI 0.78–1.11) (Table 2).

**Table 2:** Subgroup analyses and individual stain use.

Study	No. of studies	Pooled estimates		Tests of heterogeneity		
		RR (95% CI)	p value	Q value	p value	I^2^ (%)
All studies	11	0.81 (0.71–0.92)	0.002	28.18	0.002	64.5%
Study design						
Case-control	5	0.77 (0.62–0.97)	0.024	16.15	0.003	75.2%
Cohort	6	0.82 (0.68–0.99)	0.039	11.24	0.047	55.5%
Region						
North America	6	0.76 (0.54–1.08)	0.128	23.14	0.000	78.4%
Europe	4	0.86 (0.80–0.93)	0.000	2.45	0.485	0.0%
Asia	1	0.73 (0.60–0.88)	—	—	—	—
Adjusted for age						
Yes	7	0.75 (0.60–0.95)	0.016	23.20	0.001	74.1%
No	4	0.86 (0.75–0.99)	0.033	4.59	0.204	34.7%
Adjusted for gender						
Yes	7	0.76 (0.59–0.98)	0.031	26.94	0.000	77.7%
No	4	0.85 (0.79–0.92)	0.000	1.23	0.747	0.0%
Quality of studies						
High quality	7	0.71 (0.59–0.87)	0.001	17.36	0.008	65.4%
Medium quality	4	0.93 (0.78–1.11)	0.405	7.78	0.051	61.4%
Individual statin use						
Atorvastatin	4	0.83 (0.66–1.05)	0.119	9.55	0.023	68.6%
Lovastatin	2	0.61 (0.16–2.35)	0.474	5.76	0.016	82.6%
Sivastatin	4	0.68 (0.45–1.01)	0.056	75.66	0.000	96.0%
Pravastatin	3	1.35 (0.58–3.10)	0.486	6.26	0.044	68.1%
Rosuvastatin	1	0.88 (0.52–1.48)	—	—	—	—

RR, relative risk; CI, confidence interval.

### Individual statin use

Among the individual statins, atorvastatin, lovastatin, simvastatin, and rosuvastatin showed a non-significant inverse association with the risk of PD (RR 0.83, 95% CI 0.66–1.05; RR 0.61, 95% CI 0.16–2.35; RR 0.68, 95% CI 0.45–1.01; and RR 0.88, 95% CI 0.52–1.48, respectively), while pravastatin non-significantly increased the risk of PD (RR 1.35, 95% CI 0.58–3.10). These details are shown in Table 2.

### Long-term statin use

**Fig 4:**
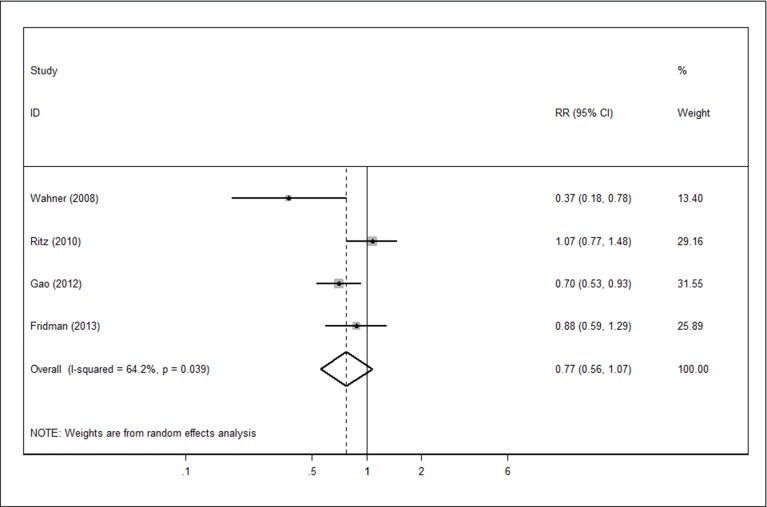
Forest plot in analysis of long-term statin use in relation to the risk of PD.

Long-term statin use decreased the risk of PD almost significantly (RR 0.77, 95% CI 0.56–1.07). However, heterogeneity was found among these studies (Cochrane Q value = 8.38, p *=* 0.039; I^2^ = 64.2%) (Fig 4).

## Discussion

A few recent studies have examined the possible association between statin use and PD. However, their findings varied widely, as noted in the introduction. Our meta-analysis combined 11 observational studies and suggested that statin use was associated with a significant decrease in PD incidence, a result that proved to be steady after the sensitivity analyses. In the overall analysis, there was moderate heterogeneity among studies, which might have been caused by differences in study design, definitions of statin use, PD diagnostic methods, and confounding variables adjusted. In addition, studies included were conducted in different regions, covering North America, Europe, and Asia, involving participants of different races, genetic backgrounds, demographic characteristics, and socioeconomic statuses. To manage these differences, we conducted subgroup analyses.

In the subgroup analyses, we obtained results similar to those for the overall analysis in two subgroups stratified by study design. When stratified by region, statins showed an inverse association with the risk of PD in all subgroups, albeit non-significantly so in studies performed in North America. The different efficacy among regions might result from the difference in race. Studies have reported variations in responses to statin therapy among races [21,22]. It is possible that the relatively greater racial diversity in North America result in a wide confidence interval in the North America group. Another two subgroup analyses showed that studies that were adjusted for age or sex, which are known to confound PD incidence, had a greater inverse association than did unadjusted studies. Subgroup analysis of high-quality studies showed an inverse association, but this relationship was non-significant in studies of medium quality. We believed that the results from adjusted analyses and high-quality subgroup more accurately reflected real effects.

As for individual statins, all statins showed a non-significant inverse association with the risk of PD, except for pravastatin. A possible explanation for the exception of pravastatin might be its different lipophilic properties. A previous study has shown that pravastatin had the weakest lipophilicity of the statins, resulting in its difficulty in crossing the blood-brain barrier and thus limiting its effect on the central nervous system [23]. According to another study, atorvastatin was the most lipophilic, followed by simvastatin [24]. However, regarding the potential to cross the blood-brain barrier, simvastatin seemed to have better access to the brain than did atorvastatin [24, 25]. This finding is in agreement with our results that atorvastatin and simvastatin had almost significantly larger protective effects. As for lovastatin and rosuvastatin, the size of the subgroup was small and could not offer a credible outcome.

The meta-analysis also revealed an inverse association between long-term statin use and risk of PD, although the relationship was not statistically significant. However, our results should be interpreted cautiously because of the limited number of studies and the different definitions of long-term exposure. In addition, long-term use might lead to poor adherence and non-continuous exposure. According to Lee, continuation of statin therapy was associated with a decreased incidence of PD as compared to discontinuation among statin users [26]. Most studies reporting an association between long-term statin use and risk of PD only determined the period of statin use without assessment of the actual continuity [9, 11, 14], which might have resulted in an underestimation of statin's effect.

The observed association between statin use and lower PD risk is consistent with the results of animal and *in vitro* experimental studies in PD models. One study reported that simvastatin effectively inhibited the activation of astrocytes, reduced TNF-α expression, and exerted anti-inflammatory effects, thus protecting the dopaminergic neurons in the substantia nigra and striatum in a rat model of PD [27]. Additional animal studies suggested that statins might provide neuroprotective effects by reducing alpha-synuclein aggregation and regulating NMDA receptors [28, 29]. Furthermore, *in vitro* studies demonstrated that simvastatin provided robust neuroprotection against dopaminergic neurodegeneration, partially via anti-inflammatory mechanisms, the PI3K/Akt/caspase 3 pathway, and by inhibiting reactive oxygen species production [30, 31].

There are several limitations in our meta-analysis. First, our results were all obtained from observational studies, which are susceptible to various biases. Selection bias, information bias, and confounding bias are inevitable in case-control and cohort studies, as well as the withdrawal bias for cohort studies. Second, pooled RRs were calculated on the basis of application of ORs, RRs and HRs as the same effect measures. Although, for diseases of low incidence (usually less than 5%) in the general population the OR and the RR are about the same and the HR could be regarded as RR containing time factors, the OR tends to overestimate the RR, albeit slightly [32]. Third, studies showed an association between lower low-density lipoprotein cholesterol (LDL-C) levels and a higher incidence of PD [5, 33]. People with high LDL-C levels were more apt to take statins compared to those with normal LDL-C levels. This difference might lead to differences in LDL-C levels between statin and non-statin groups, therefore reducing the incidence of PD among statin users because few studies adjusted for LDL-C levels. Fourth, PD cases were identified by multiple approaches without a systematic clinical examination. It was possible that some of the subjects who were diagnosed with PD actually had secondary Parkinsonism. Fifth, although an inverse association between statin use and the risk of PD was found, we could not provide any clinical recommendations about the duration and dose because of the different definitions for statin use. Finally, the conclusions regarding individual and long-term statin use might be limited, due to the small sample size.

In summary, we observed an inverse association between statin use and the risk of PD in our meta-analysis. However, our results, which were only marginally significant and could have been due to chance, should be interpreted with caution because they were all obtained from observational studies. RCTs and additional prospective cohort studies are needed to provide a convincing conclusion about the relationship between statin use and the risk of PD.

## Supporting Information

S1 File PRISMA 2009 checklist. (DOC)
